# Breast Cancer: Targeting of Steroid Hormones in Cancerogenesis and Diagnostics

**DOI:** 10.3390/ijms22115878

**Published:** 2021-05-30

**Authors:** Marcela Valko-Rokytovská, Peter Očenáš, Aneta Salayová, Zuzana Kostecká

**Affiliations:** Department of Chemistry, Biochemistry and Biophysics, University of Veterinary Medicine and Pharmacy in Košice, Komenského 73, 041 81 Košice, Slovakia; zuzana.kostecka@uvlf.sk

**Keywords:** breast cancer, steroid hormones, metabolomics

## Abstract

Breast cancer is the most common malignancy in women with high mortality. Sensitive and specific methods for the detection, characterization and quantification of endogenous steroids in body fluids or tissues are needed for the diagnosis, treatment and prognosis of breast cancer and many other diseases. At present, non-invasive diagnostic methods are gaining more and more prominence, which enable a relatively fast and painless way of detecting many diseases. Metabolomics is a promising analytical method, the principle of which is the study and analysis of metabolites in biological material. It represents a comprehensive non-invasive diagnosis, which has a high potential for use in the diagnosis and prognosis of cancers, including breast cancer. This short review focuses on the targeted metabolomics of steroid hormones, which play an important role in the development and classification of breast cancer. The most commonly used diagnostic tool is the chromatographic method with mass spectrometry detection, which can simultaneously determine several steroid hormones and metabolites in one sample. This analytical procedure has a high potential in effective diagnosis of steroidogenesis disorders. Due to the association between steroidogenesis and breast cancer progression, steroid profiling is an important tool, as well as in monitoring disease progression, improving prognosis, and minimizing recurrence.

## 1. Introduction

Breast cancer is the most common malignant neoplasm in women and is one of the leading causes of cancer deaths. The risk group consists of women over the age of 50 and the positive family history. This means several cases of breast and/or ovarian cancer in the family. Other risks of breast cancer include early age at menarche, nulliparity, first pregnancy after 35 years of age, hormonal therapies (estrogen with or without progesterone) and late menopause onset. After menopause, adipose tissue is the main source of sex hormone estrogen, and obese postmenopausal women have both higher levels of endogenous estrogen and a higher risk of breast cancer. It is well-known that breast cancer is a hormone-responsive cancer that currently accounts for about a quarter of all cancers in women [1,2].

Breast cancer is a highly heterogeneous disease, classified into five categories based on expression profiles: (I) luminal (A or B); (II) basal-like estrogen receptor negative (ER−), progesterone receptor negative (PR−) and human epidermal growth factor receptor 2 negative (HER2−), also called triple negative breast cancer (TNBC); (III) expressed human epidermal growth factor receptor 2 (HER2); (IV) normal breast-like (low expression of luminal epithelial genes and high expression of basal epithelial and non-epithelial genes); and (V) claudin-low-expressed breast cancer (low expression of cell-cell junction proteins) [3,4]. The clinical classification based on steroid hormone receptor expression is also a very useful tool for the study of breast cancer. Estrogen receptor, progesterone receptor and human epidermal growth factor receptor 2 expressions were determined as common biological markers for prognosis and survival prediction [5]. Using these markers, breast cancer can be classified more precisely into four subtypes: ER+/PR+/HER2−, ER+/PR+/HER2+, ER−/PR−/HER2+ and ER−/PR−/HER2− [6]. ER and PR are members of the sex steroid hormone receptor family, including the androgen receptor (AR); however, only ER and PR are used for cancer classification, while AR is not.

The prognosis and appropriate choice of therapy depend on the subtype of breast cancer. Estrogen, which acts through ER, is thought to be a major etiological factor in breast cancer, and currently therapies include inhibitors of the ER and an estrogen-producing enzyme, cytochrome P450 19A1 (CYP19A1; aromatase). Currently, new therapeutic strategies focus on PR and AR steroid receptors, and their ligands in various subtypes of breast cancer [7].

## 2. Steroid Hormones in Etiology of Breast Cancer

**Figure 1:**
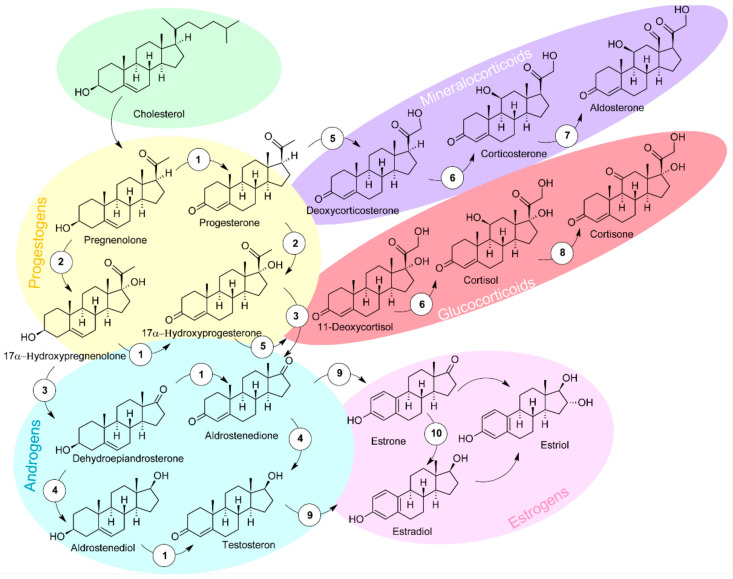
Biosynthesis and metabolism of steroid hormones. 1: 3β-hydroxysteroid dehydrogenase type 2 (3β-HSD2); 2: 17α- hydroxylase; 3: 17,20-lyase; 4: 17β-hydroxysteroid dehydrogenase type 3/5 (17β-HSD 3/5); 5: 21-hydroxylase; 6: 11β-hydroxylase; 7: aldosterone synthase; 8: 11β-hydroxysteroid dehydrogenase type 2 (11β-HSD2); 9: aromatase; 10: 17β-hydroxysteroid dehydrogenase type 1/3 (17β-HSD 1/3).

Androgens, estrogens and progestogens are sex steroid hormones that play a critical role in the etiology of breast cancer. Steroid hormones, structural derivatives of cyclopentanoperhydrophenanthrene, play a major role in modulation of many physiological processes. There are four main types of steroid hormones: progestogens, androgens, estrogens and corticosteroids, which are derived from the sterol precursor, cholesterol (Figure 1). In living systems, steroids are synthesized in the gonads, placenta and adrenal cortex. Progestogens play an important role in maintaining pregnancy and are present in other phases of the estrous and menstrual cycles. Estrogens play a key role in normal sexual and reproductive development in women and affect the endocrine, cardiovascular and metabolic systems, and bone growth [8,9].

Breast cancer tissue expresses sex steroid hormone receptors such as estrogen (ER), progesterone (PR) and androgen (AR) receptors in cancer tissue. This mechanism plays a key role in the proliferation of breast cancer cells [10]. Therefore, ER, PR and HER2 are important prognostic and predictive markers for breast cancer. Evaluation of ER, PR and HER2 status is necessary to assess tumor biology and to determine the use of hormone therapy (for ER- and/or PR-positive breast cancer) and targeted therapy with trastuzumab (for HER2-positive tumors) [11]. Due to the high heterogeneity of breast cancer, there is no impressive direct treatment for all cancer types. Triple-negative breast cancer (TNBC) lacks the expression of ER, PR and HER2, which are the objectives in targeted therapies.

So, it is important to develop new therapeutic strategies as alternatives to currently used drugs such as tamoxifen, trastuzumab or lapatinib [12,13].

**Figure 2:**
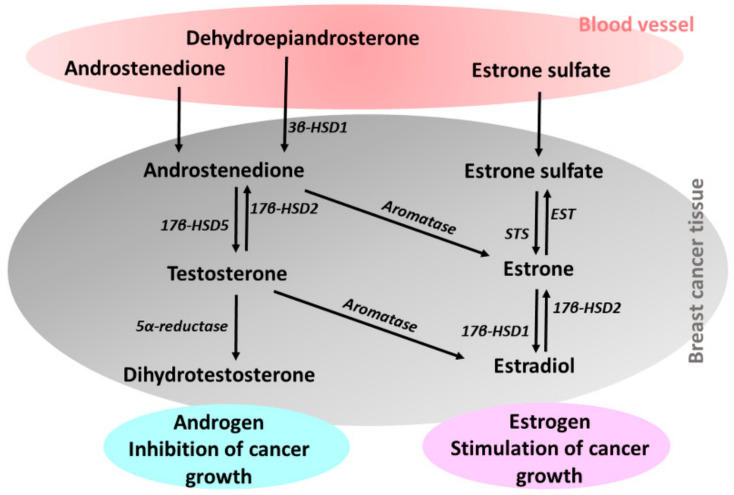
Schematic of steroid metabolism in breast cancer tissue. The bioactive steroids dehydroepiandrosterone, androstenedione and estrone sulfate are formed from circulating inactive steroids by enzymes in steroid metabolism. STS, steroid sulfatase; EST, estrone sulfotransferase; 3β-HSD1, 3β-hydroxysteroid dehydrogenase type 1; 17β-HSD1, 17β-hydroxysteroid dehydrogenase type 1; 17β-HSD2, 17β-hydroxysteroid dehydrogenase type 2; 17β-HSD5, 17β-hydroxysteroid dehydrogenase type 5. Modified and redrawn according to Suzuki et al. [17].

Several studies have shown that tumor tissues have local steroid synthesis [14,15]. Biologically active estrogens are synthesized by estrogen-producing enzymes, such as aromatase, which converts circulating androstenedione to estrone or testosterone to estradiol; steroid sulfatase (STS), which hydrolyzes of circulating estrone sulfate to estrone; and 17β-hydroxysteroid dehydrogenase type 1 (17β-HSD1), which converts estrone to estradiol in breast cancer (Figure 2) [16].

Intratumoral androgen concentrations have also been reported to be significantly higher in breast cancer, along with androgen-producing enzymes such as 17β-hydroxysteroid dehydrogenase type 5 (17β-HSD5), which converts circulating androstenedione to testosterone, and 5α-reductase, which reduces testosterone to dihydrotestosterone, have been expressed [17].

Circulating estrogens are primarily excreted from the ovaries in premenopausal women, but many invasive breast cancers develop after menopause when the ovaries cease to function. High intratumoral estradiol levels in postmenopausal women are thought to be maintained by intratumoral estrogen biosynthesis [18]. Intracellular estradiol is formed from two sources, namely, peripheral estradiol by transport into the tumor cell and intratumoral biosynthesis from other steroids. It is well known that cytosolic receptor levels are always low in tumors in premenopausal women with high serum estrogens, due to blockade of the receptor by endogenous estradiol. In postmenopausal women, tumors are low in peripheral estrogen as a result of intratumoral estradiol biosynthesis. Intratumoral levels of estrogens have also been found to correlate with the tumor gene expression of metabolising enzymes and the estrogen receptor gene [19]. Among estrogen-producing enzymes, aromatase is the most important enzyme. Aromatase as the key estrogen synthase converts androstenedione and testosterone to estrone and estradiol. Aromatase is often expressed in human breast cancer tissues. Aromatase inhibition is the most effective endocrine treatment of postmenopausal women with ER-positive breast cancer [20].

Study by Miller et al. [21] demonstrated that the concentration of the biologically active estrogen, estradiol, was significantly higher in breast cancer tissue than in plasma, and intratumoral estradiol levels did not differ significantly in patients with premenopausal and postmenopausal breast cancer. Extragonadal estrogen secretion contributes to the progression of postmenopausal breast cancer. Breast tissue expresses aromatase, which mediates the conversion of circulating androgen precursors derived primarily from the adrenal glands to estradiol. After menopause, estradiol is a growth stimulus for approximately 70% of ER-expressing breast cancer cells [22].

Steroid hormones are known to diffuse freely from serum across the phospholipid membrane of cells [23]. The study by Chetrite et al. [24] confirmed that estradiol levels were significantly higher in tumor tissue than in breast tissue considered normal. Invasive postmenopausal breast cancers often express estrogen receptor, therefore local estrogen production plays an important role in the proliferation of invasive breast cancer cells in postmenopausal women [25].

Androgens, in contrast to estrogens, have predominantly antiproliferative effects via androgen receptors on breast cancer cells. In most cases of breast carcinoma (90%), ARs are expressed. AR expression is considered a positive prognostic factor for ER-positive breast cancer [26,27].

The relationship between circulating androgen levels and the risk of breast cancer is still unclear. Circulating androgens in women are dehydroepiandrosterone sulfate, dehydroandrostenedione, androstenedione, testosterone and dihydrotestosterone.

Circulating androgens are detected in premenopausal and postmenopausal women with various concentrations. Testosterone levels begin to decline in the middle reproductive years, and adrenal androgenic steroid levels (androstenedione and dehydroepiandrosterone) decline throughout postmenopausal life. Even though androgen levels decrease during menopause, this change is not as significant as a decrease in circulating levels of estrogen and progesterone due to decreased ovarian function. Although this reduces estrogen and progesterone production, synthesis of testosterone continues at constant levels and, to a lesser extent, that of androstenedione does too [28].

It has been suggested that AR could be an emerging therapeutic target in breast cancer, especially tumors that are ER-negative, and scientific interest has been focused on the clinical significance of AR expression in ER-positive or triple-negative breast cancer [29,30,31]. In a study by Choi et al., it has been suggested that AR-positive TNBCs represent a specific subset of breast cancer with poorer clinical outcome, and AR blockade could be a potential endocrine therapy for patients with AR-positive TNBCs [11].

## 3. Steroid Metabolomics in Breast Cancer

Metabolomics, the latest member of the “omics” family, focuses on the identification and quantification of metabolites, such as amino acids, carbohydrates and steroids that are found in biological systems. The analysis of the metabolome provides comprehensive information on potential biomarkers from biological samples, such as blood, tissue, saliva and urine, with relatively easy sample preparation and high-throughput [32].

Early diagnosis and monitoring are very important to improve the survival rate of breast cancer patients [33]. Currently, tissue biopsy is the gold standard for accurate diagnosis of breast cancer, but it is not suitable for routine clinical purposes. The development of a simple and feasible method for diagnosing and monitoring breast cancer has therefore become an urgent need for breast cancer research [34].

Many studies on steroidomics in clinical diagnosis and doping control have been performed in recent [35,36,37]. Anh et al. [38] investigated the role of steroidomics in the prevention, evaluation and treatment of cancer and showed that various types of steroids are significantly associated with common cancers, especially breast, endometrial and prostate cancers.

Especially, estrogens and estrogen metabolites are closely associated with breast cancer. The mechanism of estrogen-related enhancement of breast cancer incidences remains unclear, besides that two mechanisms are usually considered. In breast cancer formation *de novo*, estrogens can participate though either receptor-dependent or -independent mechanisms. First is the ER action-mediated stimulation of breast cells proliferation and rate of mutations, and the second, metabolism of estradiol to genotoxic metabolites in breast tissue and consequent increase in DNA mutations [39,40,41].

Breast cancers as hormone-sensitive or hormone-receptor-positive cancers are characterized by increased estrogen production [42]. Biotransformation of estrone and estradiol leads to the formation of catechol derivatives such as 4-hydroxyestradiol, 2-hydroxyestradiol, 2-hydroxyestrone and 4-hydroxyestrone, which are presumed to be carcinogenic metabolic products, whereas 2-methoxyestrogens are presumed to be anti-proliferative and protective against carcinogenesis [43].

For other differentially expressed steroids, the most significant of the steroid biomarkers were estradiol, dehydroepiandrosterone and cortisol. Anh et al. [38], in their study, pointed to significant changes in steroidogenesis, androgen and estrogen metabolism, and androstenedione metabolism in cancers. Their findings suggest that estradiol, dehydroepiandrosterone, cortisol and estrogen metabolites may be considered as oncosteroids. In particular, estradiol has been associated with an increased risk of breast and ovarian cancer, but with a decreased risk of esophageal/gastric cancer [44,45,46]. Other steroids, such as testosterone, androstenedione and dehydroepiandrosterone sulfate, have also been studied to predict an increased risk of breast cancer in premenopausal women with elevated blood concentrations of androgens [47]. Pasanisi et al. [48] also reported increased serum testosterone levels in the presence of metabolic syndrome, which was closely related to breast cancer progression. Testosterone may be more strongly associated with risk of ER+ breast cancer as well as estradiol, but this relationship was not statistically significant [49].

## 4. Analytical Tools of Steroidomics

**Figure 3:**
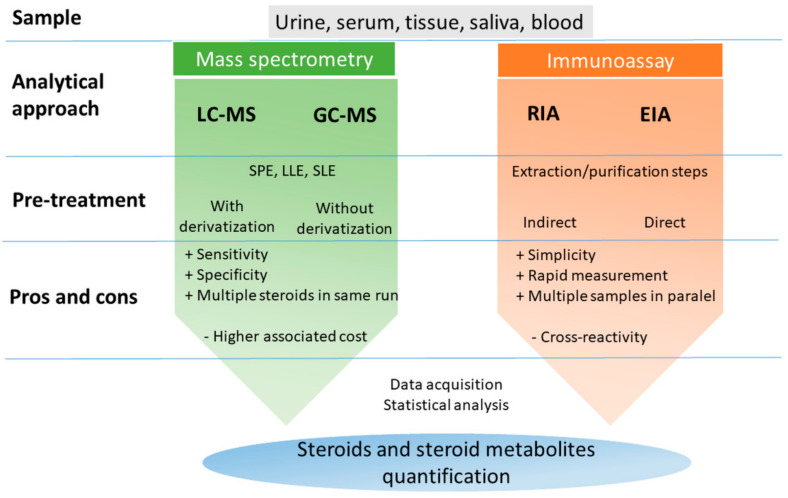
The main techniques for steroids determination in different biological samples. LC-MS—liquid chromatography-mass spectroscopy; GC-MS—gas chromatography-mass spectrometry; RIA—radioimmunoassay; EIA—enzyme immunoassays; SPE—solid-phase extraction; LLE—liquid–liquid extraction; SLE—supported liquid extraction.

Accurate, sensitive measurement of steroid hormones in biological fluids is a fundamental pillar of mechanistic understanding in modern reproductive biology and medicine. Different analysis methods have been developed to determine steroid hormones, including immunoassay (radioimmunoassay RIA, enzyme immunoassay EIA), mass spectrometry (MS), gas chromatography (GC) and high-performance liquid chromatography (HPLC) [50,51,52]. Immunoassay, first developed in 1959 [53], is nonspecific and insensitive method, which easily leads to cross reactivity, and multiple steroid hormones cannot be simultaneously determinate [54]. The main techniques for the determination of steroids in various biological samples are shown in Figure 3.

Most reported direct radioimmunoassay methods showed a limit of quantification of 30 to 100 pg/mL, which is not suitable for the analysis of estrogens in the plasma of postmenopausal women (1–10 pg/mL) [55,56]. Similarly, there is a limitation for the determination of testosterone by immunoassays due to background interference with dehydroepiandrosterone sulfate, which is present in abundant concentrations in human serum [57].

On the other hand, steroid measurement by MS has been available even longer than immunoassays for steroids [58] and as a chemical analytical method based on the structural properties of steroids has long been considered the gold standard reference method for steroid specificity. Technological advances have resulted in MS equipment integrated with LC that retains the specificity of the reference level and corresponds to or exceeds the sensitivity of immunoassays [52].

Some analytical methods associated with breast cancer diagnostic are mentioned in the overview Table 1.

Currently, LC-MS and GC-MS techniques are the methods of choice for estrogen analysis, but GC-based techniques are limited by the use of large sample volumes. GC-MS analysis of sterols and steroids was first used in 1960 and marked the beginning of development for the practical use of mass spectrometry in endocrine studies [58,67]. Though GC-MS can significantly increase the specificity for the measurements of steroids, the detection sensitivity is limited, and the sample preparation is time-consuming [68]. Unfortunately, not all steroid compounds possess ionizable groups, resulting in poor GC-MS sensitivity in ESI mode, therefore derivatization step is also necessary [69].

The measurement of large numbers of sex steroids in clinical epidemiology and laboratory research using reliable methods providing low limits of quantification and the use of a limited volume of blood sample is still a challenge. Caron et al. reported a highly sensitive and specific GC-MS/MS method for the simultaneous quantification of ten endogenous steroids (progesterone, dehydroepiandrosterone, androstenediol, androstenedione, testosterone, dihydrotestosterone, androsterone, 5α-androstane-3β,17β-diol, estrone and estradiol) using only 250 µL of serum. The method was also used to detect estradiol in the low pg/mL range (LLOQ 1 pg/mL) [65].

A number of analytical methods have already been used and developed to provide specific descriptive and quantitative information concerning endogenous estrogens and their metabolites. For example, high-performance liquid chromatography with electrochemical detection (HPLC-ECD) has been used to determine estrogens and their metabolites in animal urine and human tissue [70,71]. However, the ECD did not provide sufficient sensitivity for clinical analysis and there was background interference due to the complexity of the biological samples [72].

Currently, the fragmentation pathways of several steroid hormones have been investigated by ultra-high performance liquid chromatography tandem MS (UHPLC-MS). A recent study by Zheng et al. presented an integrated method of human urine analysis based on UHPLC with high-resolution mass spectrometry in a data-dependent data acquisition mode followed by a parallel reaction monitoring mode. The proposed method made it possible to characterize a total of 80 and 107 steroidal hormones in human urine of men and women, respectively [73].

For LC-MS methods, it is necessary to select reaction monitoring based on tandem mass spectrometry (MS/MS). Interpretation of the product ion spectra of steroid hormones and investigation of their collision-induced metabolic pathways are important for the mass spectrometric characterization of new “designer” steroids. This is useful for understanding the specificity of steroid product ions, as well as for detecting, identifying and confirming them [74].

Owing to its high sensitivity, specificity, accuracy and reproducibility, multiple reaction monitoring has been developed as the method of choice for quantitative and qualitative determination of endogenous estrogens and their metabolites in human serum [75] and human urine [72].

Chemical derivatization is a general sample pretreatment process that modifies the functional groups of analytes via the reactions using derivative reagents to augment the UV, fluorescence or mass spectrometric response of the analytes. In order to increase the detection sensitivity, a derivatization step is required prior to analysis [69]. Liquid chromatography linked with electrospray ionization (ESI) tandem mass spectrometry (MS/MS) is the most upgraded technique for steroid analysis, but its absolute sensitivity is still analyte-dependent. One of the most effective methods to overcome the sensitivity problem is chemical derivatization [76].

As was mentioned, the sensitivity of the analytical method due to the low concentration of steroid in biological samples plays the crucial role. Appelblad et al. characterized the new dansylation reaction with trifluoromethanesulfonic acid as catalyst for five ketosteroids (progesterone, 5α-pregnane-3,20-dione, 5β-pregnane-3,20-dione, 3α-hydroxy-5β-pregnan-20-one and 3α,21-dihydroxy-5β-pregnan-20-one). The simple and sensitive analysis of dansylhydrazones of ketosteroids can be used for qualitative and quantitative determination. The corresponding LOD values obtained from the calibration data were 12 and 15 pmol (*n* = 4; 500 µL injected) [77]. Determination by high-performance liquid chromatography with fluorescence detection of progesterone, 17-hydroxyprogesterone and four other 3-ketosteroids was studied in Katayama’s research group. All steroids were modified with 4,4-difluoro-5,7-dimethyl-4-bora-3a,4a-diaza-s-indacene-3-propionohydrazide (BODIPY FL hydrazide), a derivatized agent. LOD (progesterone, 17-hydroxyprogesterone, dehydroepiandrosterone, androstenedione, testosterone and 17-methyltestosterone) were 550–3700 fmol per 10 µL injection serum. [78]. The suggested HPLC method was the most sensitive among reported methods [77,79,80] with fluorescence and chemiluminescence derivatization with dansylhydrazine.

Fiers et al. reported an LC-MS/MS method for the assay of estrone and estradiol in human serum without derivatization, and the limit of quantification (LOQ) was below 0.5 pg/mL [81]. Khedr and Alahdal, in their work, described an enhanced sensitive LC-ESI-MS/MS method that is quantifying estrogen and its metabolites in plasma of healthy postmenopausal women and women with diagnosed breast cancer. Presented method was used to analysis of ten estrogen metabolites with a previous derivatization step, and limits of detection (LOD) were 0.1–0.8 pg/mL plasma. The average plasma level of 4-hydroxy-estrone in breast cancer was greater by 51-fold than in healthy cases. The catechol estrogens concentrations (4-hydroxyestradiol, 2-hydroxyestradiol and 16α-hydroxyestrone) in breast cancer were higher by 5–11-fold in comparison with healthy cases. The concentrations of methoxyestrogen metabolites (2-methoxyestrone, 4-methoxyestradiol and 2-methoxyestradiol) were also higher in cancer cases (2–10-fold). The free estrogens (estrone, estradiol and estriol) concentrations were approximately within the normal range [43]. Laforest et al. developed a new assay of estrogens and glucocorticoids by LC-MS/MS coupled with derivatization to allow simultaneous quantification of a panel of steroids in human breast adipose tissue across the endogenous range of concentrations encountered in health and disease. The method was applied to samples from healthy women undergoing reduction mastectomy and breast cancer patients undergoing partial mastectomy. They were able to detect and quantify estrogens in more than 90% of samples using around 200 mg of adipose tissue. Cortisol was detected in all breast adipose tissue samples and cortisone in most [61]. Lee et al. developed analytical method to determine the estrogens levels in hair samples collected from 10 healthy females (age: 26.2 ± 1.0 years) by liquid chromatography electrospray ionization tandem mass spectrometry (LC-ESI-MS/MS) using derivatized samples with dansyl chloride to increase sensitivity [82]. Qin et al. studied the simultaneous analysis of carbonyl and phenolic hydroxyl-containing steroid hormones (estrogens, androgens, corticoids and progestogens). This method was used to study steroid hormones in relation to ovarian cancer, and significant changes were shown in serum of patients. Twenty-nine steroid hormones were detected at pg/mL or ng/mL (two hormones) levels after derivatization in serum samples. Progesterone, cortexolone, hydrocortisone, estradiol, estriol, testosterone, 4-androstene-3,17-dione, 5α-tetrahydrocorticosterone, etiocholanolone, epitestosterone, allotetrahydrocortisol and dehydroepiandrosterone sulfate in serum samples showed a significant difference (*p* < 0.05) between healthy female and ovarian cancer patients [83]. Solheim et al. studied oxysterols as cholesterol metabolites that can be formed by enzymatic or nonenzymatic oxidation. In breast cancer, 27-hydroxycholesterol is enzymatically created that potentiates cell proliferation and tumor growth via its ability to bind and activate the ER. Esterified and free hydroxylated oxysterols levels were determined in breast cancer tumors using a novel fast liquid chromatography tandem mass spectrometry method. In ER-positive tumors, a strong correlation between free and esterified 27-hydroxycholesterol (27-OHC) and 25-hydroxycholesterol (25-OHC) was observed, suggesting comprehensive metabolism oxysterol metabolism pathways in these tumors. On the contrary, in the case of ER-negative tumors only weak (for 27-OHC) or absent correlations (24S-OHC and 25-OHC) indicate different rolls in more aggressive type. 27-OHC, a selective ER modulator, is considered as marker in metastasis and proliferation of ER positive breast cancer. Similarly, it can initiate the liver X receptor (LXR, a member of the nuclear receptor family of transcription factors) and play a key role in metastatic and/or chemotherapy-resistant triple-negative breast cancer [60].

MCF-7 is the most studied ER-positive breast cancer cell line in the world and has the most valuable influence to breast cancer research. Metabolomic study of steroids level in this estrogen-dependent tumor cell line was undertaken by Poschner et al. using LC-MS. Within this study, MCF-7 tumor cells were treated with hormone precursor estrone and dehydroepiandrosterone and monitored the formation of their metabolites. According to this method, the monitoring of the estrogen metabolites pathway has been provided. When dehydroepiandrosterone was administered; three metabolites, namely, dehydroepiandrosterone sulfate, androstenedione, and testosterone, were detected due to low aromatase expression in MCF-7 cells. On the contrary, incubation of estrone induced the formation of metabolites estradiol; 16α-hydroxy-17β-estradiol; and the conjugated estrogen metabolites estrone-3-sulfate, 17β-estradiol-3-(β-D-glucuronide), and 17β-estradiol-3-sulfate, and the level of estrone decreased with time [62].

Determination of steroid conjugates in biological matrixes has been described in different literatures [84,85,86,87]. Human endogenous compounds are responsible for biotransformation reactions that result in the formation of metabolites with different chemical structures, physiological, pharmacological or toxicological properties compared to their parent compounds. The LC-MS/MS method allows for the direct analysis of steroid conjugates without prior hydrolysis. The use of LC-MS/MS has led to the identification of previously unreported conjugate metabolites for some exogenous and endogenous steroids, such as sulfates or conjugates with cysteine and *N*-acetylcysteine. Glucuronidation is one of the most important phase II metabolic reactions during the biotransformation of endogenous steroids. Glucuronidation is generally a detoxification pathway because glucuronoconjugates are less active and more soluble in water than the parent compound, which facilitates their excretion from the body. On the other hand, for some compounds such as 17β-hydroxyestrogens, testosterone and dihydrotestosterone, higher toxicity was observed for glucuronide conjugates compared to the corresponding parent compounds. Fabregat et al. developed and evaluated liquid chromatography-tandem mass spectrometry (LC-MS/MS) scan methods for the open detection of steroid glucuronides (6β-hydroxyandrosterone, 6β-hydroxyetiocholanolone) in urine samples [88].

As early as the 1980s, several studies focused on the ratio of 2-hydroxyestrone to 16α-hydroxyestrone (2-hydroxylation vs. 16α-hydroxylation), which is closely related to the higher risk of breast cancer [89,90]. This hypothesis is based on data from studies on the tumorigenic and genotoxic effect of 16α-hydroxyestrone and the protective effect of 2-hydroxyestrone. This effect of 16α-hydroxyestrone results from the possibility to form covalent adducts with macromolecules [91]. On the other hand, this hypothesis has not been proven despite many studies. The main problem was the lack of precision in used methodologies, such as RIA and EIA, as well as LC-MS/MS. This limitation results from the determination of unconjugated 2-hydroxyestrone, 2-hydroxyestradiol and 16α-hydroxyestrone in serum or intact sulfated and glucuronidated estrogen metabolites in serum or urine [92].

As already mentioned, the increased risk of breast cancer is closely related to an imbalance in estrogen metabolism. Van der Berg et al. developed an analytical method for the quantification of 27 estrogen-related metabolites in urine by LC-ESI-MS/MS. Quantification of metabolites by the proposed method included parent estrogens, hydroxylated and methylated forms, metabolites of the 16α-hydroxyestrogen pathway, sulfate and glucuronide conjugated forms, precursors and related steroid hormone. The analysis required purification of the sample by a multi-step solid phase extraction procedure and derivatization of the metabolites by dansylation [93].

## 5. Perspective of Metabolomics in Clinical Application

Metabolomics is an analytical approach using the systematic determination of metabolite profiles and diseases. Common analytical techniques applied in metabolomics are nuclear magnetic resonance (NMR), GC-MS and LC-MS. Advances in analytical technologies have made it possible to quickly determine the levels of thousands of metabolites in any biosample, leading to the use of metabolomics analysis in various clinical studies [94].

Disease biomarkers detection plays an important role not only in early diagnosis but also in classification or assessment prognosis and treatment response. Research metabolomics is based on statistical comparisons between groups to identify areas of metabolism that differ or to find biomarkers that are different between groups [95].

Nowadays, applications of metabolomics methods are becoming apparent in clinical testing. Using LC-MS/MS belongs to well-accepted technology and is increasingly replacing problematic immunoassay methods due to its greater sensitivity and specificity. However, several aspects of its application differ as clinical tests vary from applications focused on research or new biomarker discovery and for purpose must be clinically validated [95].

Besides that, steroid analysis by LC-MS/MS in daily clinical routine diagnostics requires conditions with high-throughput, including rapid chromatographic separation. Gaudl et al. presented a high-throughput method, LC-MS/MS/MS (LC-MS^3^), for steroid analysis and implemented it in a clinical routine laboratory. This method allowed for the simultaneous quantification of 17α-hydroxyprogesterone, aldosterone, androstenedione, cortisol, cortisone, dehydroepiandrosterone sulfate, estradiol, progesterone and testosterone in human serum, urine, saliva and hair [96]. In 2019, the same authors investigated strategies for optimizing methods to increase specificity for serum cortisol and 17α-hydroxyprogesterone in dried blood. In their study, they investigated improved chromatography, ionization polarity switching and detection through two-step fragmentation (MS^3^) using a quadrupole linear ion trap [97].

Prostate and adrenal cancer are the two most investigated diseases related to steroids, followed by breast and endometrial cancer. The roles of steroids were also explored in other types of cancer, such as ovarian, esophageal, gastric, and liver cancer. Regarding the sample type, serum, urine and plasma are the three types of biospecimen used in studies concerning steroid biomarkers. Changes in estrogen and androgen metabolism were associated with endocrine-related cancers, including prostate, breast, endometrial and ovarian cancer. Significant increases of estradiol, estrone, 2-methoxyestrone, 2-hydroxyestrone, estriol, 16-epiestriol, 16α-hydroxyestrone, etiocholanolone, 16-ketoestradiol, 2-hydroxyestrone-3-methyl ether, 4-hydroxyestrone and 17-epiestriol, and decrease of androsterone in breast cancer, were monitored. Oncosteroidomics is a targeting method for the future clinical application. Next, it will be necessary to establish standardized methods to employ the modifications of steroid metabolic networks for the prevention, assessment and management of cancers [38].

The introduction of steroid tests into routine practice has increased. Some diagnostic kits for measuring steroids are available, but their cost-effectiveness in normal use has yet to be seen. LC-MS/MS methods are becoming easier to use and are more robust but require sufficient analytical skills. This is an issue of laboratory training, and the LC-MS/MS service can be maintained in a routine laboratory [98].

## 6. Conclusions

Among types of cancer with the highest incidence is breast cancer. A high percentage of women die from the disease each year as a result of late diagnosis associated with neglected or inadequate prevention, when the cancer is already advanced and treatment options are minimized.

The basis of the success of breast cancer therapy and increasing the survival rate of patients is its early and accurate diagnosis. In modern medicine, the most rapid and least invasive diagnostic methods are introduced. This review focused on the steroid metabolomics of breast cancer, which represents a huge potential in diagnostics aimed at studying the key metabolic pathways associated with carcinogenesis.

## Figures and Tables

**Table 1:** Outline of analytical strategies based on steroidomics as tools in breast cancer diagnostics.

Method	Derivatization	Steroids Quantified	Sample	Sensitivity LLOQ	Ref.
LC-ESI-MS/MS	DNSCl	16 estrogen metabolites	86 postmenopausal female breast cancer patients’ urine and 36 healthy controls	2 pg/mL	[59]
LC-MS/MS	Girard T reagent	free and esterified oxysterols	ER-positive (*n* = 11), ER-negative (*n* = 11) breast cancer tissue	15 pM–31 pM	[60]
LC-ESI-MS/MS	PPZ and methyl iodide (estrogens)	estradiol, estrone, cortisone and cortisol	adipose tissue from women with and without breast cancer	15 and 100 pg per sample	[61]
LC-HRMS	-	10 steroids and metabolites of estrogenic pathway	MCF-7 breast cancer cell culture	0.005–2 ng/mL	[62]
LC-ESI-MS	MNAHS	16 estrogens and their metabolites	breast cancer serum	LOD 0.36–2.3 ng/mL	[63]
GC-MS/MS	PFPA (androgens/progestins) ECF + PFPA (estrogens)	15 estrogens 6 androgens 2 progestins	16 postmenopausal breast cancer tissue	LOQ 0.180–1.25 pg	[64]
GC-MS/MS	PFB-NH_2_ * PFB-Cl **	10 endogenous steroids	twenty clinical serum samples from healthy premenopausal women (*n* = 10), healthy postmenopausal women (*n* = 20) and fifteen healthy men (*n* = 15)	1–100 pg/mL	[65]
RIA	-	androstenedione, testosterone, estrone and estradiol	20 breast adipose tissues /10 breast tumors	-	[66]

LC-MS/MS—liquid chromatography-tandem mass spectrometry; UFLC-MS/MS—ultra-fast liquid chromatography-tandem mass spectrometry; GC-MS/MS—gas chromatography-tandem mass spectrometry method; DNSCl—dansyl chloride; PFB-Cl—pentafluorobenzoyl chloride; PFB-NH_2_—pentafluorobenzoyl amine; PPZ—1-(2,4-dinitro-5-fluorophenyl)-4-methylpiperazine; MNAHS—N-methylnicotinic acid N-hydroxysuccinimide ester; PFPA—pentafluoropropionic anhydride; ECF—ethyl chloroformate * androstenedione, progesterone ** androsterone, androstenediol, dehydroepiandrosterone, testosterone, dihydrotestosterone, 5α-androstan-3β-17β-diol, estrone and estradiol.
